# Management of Moderate to Severe Psoriasis in Patients with Metabolic Comorbidities

**DOI:** 10.3389/fmed.2015.00001

**Published:** 2015-01-21

**Authors:** Paolo Gisondi, Arturo Galvan, Luca Idolazzi, Giampiero Girolomoni

**Affiliations:** ^1^Section of Dermatology and Venereology, Department of Medicine, University of Verona, Verona, Italy; ^2^Section of Rheumatology, University of Verona, Verona, Italy

**Keywords:** psoriasis, comorbidities, conventional therapies, biological therapies

## Abstract

Psoriasis is a chronic inflammatory skin disease affecting 2–3% of worldwide population. The extent of skin involvement is variable, ranging from a few localized plaques to generalized involvement. Moderate to severe psoriasis (>10% of body surface area) is frequently associated with psoriatic arthritis and metabolic diseases, like abdominal obesity, diabetes, non-alcoholic fatty liver disease, dyslipidemia, metabolic syndrome, and chronic kidney disease. A common genetic background as well as several acquired risk factors links psoriasis to comorbidities. From a clinical prespective, the understanding of the patients in the context of these comorbidities is very important to ensure that treatment is tailored to meet the individual patient needs. Indeed, some pharmacological treatments may negatively affect cardio-metabolic comorbidities, and have important interactions with drugs that are commonly used to treat them. Non-pharmacological intervention such as diet, smoking cessation, and physical exercise could both improve the response to treatments for psoriasis and reduce the cardiovascular risk.

## Introduction

Psoriasis is an immune-mediated chronic inflammatory disease affecting approximately 2–3% of Caucasian population (1). It can occur at any age, although the majority of cases develop before the age of 40 years and it is uncommon in children. Psoriasis is a complex disease with a strong genetic background (1). So far, about 20 chromosome regions have been proposed to harbor psoriasis susceptibility genes, which affect mostly innate and adaptive immune responses. The locus carrying the highest risk is the class I region of the major histocompatibility antigen cluster, which harbor the human leukocyte antigen Cw6 and is associated to early onset psoriasis (2). The current understanding of the molecular pathogenesis of psoriasis assigns central importance to the interaction between acquired and innate immunity (3). Chronic plaque psoriasis is the most common type of the disease (4). The extent of skin involvement is widely variable, ranging from a few localized plaques at extensor sites to generalized involvement. Moderate to severe psoriasis is defined if the body surface involvement is >10%. Patients with psoriasis, like those with other major medical disorders, have a worse quality of life, reduced life expectancy as well as employment and income (5).

## Chronic Plaque Psoriasis and Metabolic Comorbidities

Several epidemiological studies have confirmed that moderate to severe psoriasis is strongly associated with cardio-metabolic disorders including hypertension, obesity, type 2 diabetes, dyslipidemia, non-alcoholic fatty liver disease (NAFLD), metabolic syndrome, and chronic kidney disease (CKD) (6). In particular, patients with psoriasis are more frequently overweight or obese than the general population, and the severity of psoriasis is correlated to body mass index (BMI) (7, 8). The association between obesity and psoriasis has been recently confirmed also in pediatric patients (9). Obesity generally precedes the development of psoriasis and the BMI is correlated to an increased risk of incident psoriasis. Several measures of adiposity, including BMI, waist, and hip circumference and waist–hip ratio have been reported as independent risk factors for the development of psoriasis and psoriatic arthritis (PsA) (10). The relationships between psoriasis and obesity may be largely explained by the complex properties of the adipose tissue. Indeed, the adipose tissue is not only a storage fat organ but an active endocrine organ with many secretory products, such as free fatty acids, adipocyte-derived hormones, and pro-inflammatory adipokines, including chemerin, resistin, visfatin, interleukin (IL)-6, and tumor necrosis factor (TNF)-α (11). In addition, a recent meta-analysis reported that psoriasis is a risk factor for both prevalent and incident type 2 diabetes mellitus (12, 13).

Visceral obesity and type 2 diabetes are two main components of the metabolic syndrome. The metabolic syndrome is a set of metabolic disorders, in particular insulin resistance, which may confer a higher pro-inflammatory and pro-thrombotic risk. The diagnosis of metabolic syndrome is confirmed in presence of three or more of the following conditions including abdominal obesity (waist circumference >102 cm in men; >88 cm in women), elevated serum triglycerides [ >150 mg/dl (1.7 mmol/l) or treatment], low HDL cholesterol [men <40 mg/dl (1 mmol/l); women <50 mg/dl (1.3 mmol/l); or treatment], elevated blood pressure (>130/85 mmHg or treatment), and elevated fasting glucose (>100 mg/dL or treatment) (14). In a cross-sectional study, we found that patients with psoriasis had a higher prevalence of metabolic syndrome than those with other inflammatory skin diseases after controlling for sex and age (30.1 vs. 20.6%, OR: 1.65, 95% CI 1.16–2.35) (14). The association between psoriasis and metabolic syndrome has been also recently confirmed in a meta-analysis showing that psoriasis carries a pooled odds ratio for metabolic syndrome of 2.26 (95% CI 1.70–3.01) (15). NAFLD is a spectrum of progressive liver disease that encompasses simple steatosis, non-alcoholic steato-hepatitis (NASH), fibrosis, and ultimately cirrhosis. NAFLD is recognized as the hepatic expression of the metabolic syndrome, as these conditions have insulin resistance as a common pathophysiological mechanism (16). We found that the prevalence of NAFLD in patients with chronic plaque psoriasis was remarkably greater than that in non-psoriasis controls (47 vs. 28%; *p* < 0.0001) who were matched by age, sex, and BMI (17). In a recent Dutch study, NAFLD was diagnosed in 46.2% of patients with psoriasis compared with 33.3% of the controls (*p* = 0.005) (18). Psoriasis was found associated to NAFLD independently of alcohol consumption, smoking status, presence of metabolic syndrome, and serum levels of alanine aminotransferase (adjusted odds ratio 1.7, 95% CI 1.1–2.6) (18). NAFLD is an emerging risk factor for cardiovascular diseases (CVD) (19). There is conflicting evidence as to whether psoriasis is associated with increased cardiovascular outcomes. Patients with psoriasis show a higher prevalence of other important CVD risk factors, including smoking, sedentary habit and dyslipidemia, making them more susceptible to development of CVD morbidity and mortality, and particularly young patients with the more severe skin disease (20, 21). Some studies reported that patients with severe psoriasis, but not those with mild disease, are at increased risk of developing acute myocardial infarction and stroke, as well as CVD mortality (22). Uncertainty still remains about whether the increased CVD risk observed in psoriatic patients is directly attributable to psoriasis itself or to the effects of multiple co-existing risk factors (23).

Recently, the association between psoriasis and CKD has also been reported (24). A retrospective cohort study showed in that moderate to severe psoriasis is associated with CKD independently of traditional risk factors. In particular, it was found that 5% of patients with psoriasis had CKD compared to 2% of controls. No association was shown in patients with mild disease (with a body surface area <2%). The relative risk of CKD was observed being especially increased in younger patients. Nerveless, despite the attenuation of the association with increasing age, the clinical relevance of the absolute risk of CKD attributable to psoriasis was demonstrated increasing with age. Because of these findings closer monitoring for renal insufficiency, such as routine screening for microalbuminuria and serum creatinine should be performed in patients with moderate to severe disease. Additionally, the risk vs. benefit of potentially nephrotoxic drugs such as cyclosporine in patients with moderate to severe psoriasis should be carefully considered.

The association between psoriasis and comorbidities could be explained considering a common genetic background, the systemic effects of chronic inflammation, insulin resistance, and an unhealthy life-style such as heavy smoking/drinking, over-eating habit, and sedentary life, which are common in patients with psoriasis. A meta-analysis of 4 different studies reported that 10 metabolic gene single nucleotide polymorphisms including those related to CARD14 are closely correlated to both psoriasis and metabolic disorders, such as diabetes, dyslipidemia, and hypertension (25). Psoriasis and obesity-related inflammatory status can fuel reciprocally. Indeed, it is likely that cytokines released from either psoriatic keratinocytes or inflammatory cells infiltrating psoriatic skin might induce systemic insulin resistance, thus favoring the development of type 2 diabetes mellitus (11). On the other hand, it is also possible that the co-existing metabolic comorbidities might directly contribute to exacerbate psoriatic inflammation through the release of several pro-inflammatory mediators from the liver and/or visceral adipose tissues, such as increased reactive oxygen species, C reactive protein (CRP), IL-6, and other adipokines (26).

## Management of Patients with Moderate to Severe Psoriasis and Metabolic Comorbidities

The association between psoriasis and cardio-metabolic disorders has important clinical consequences. Firstly, systemic treatments for psoriasis could negatively affect metabolic comorbidities, especially in case of continuous and prolonged use (Table 1). In particular, methotrexate should be prescribed with caution in the presence of obesity, diabetes, NAFLD, and heavy alcohol intake because of the increased risk of liver fibrosis (27). Transient elastography and FibroTest are effective non-invasive tools for monitoring hepatotoxicity in patients receiving methotrexate for psoriasis (28). Moreover, it should be considered that CKD of older patients could reduce renal clearance of methotrexate favoring toxicity (29). Cyclosporine can induce or worsen arterial hypertension, increase insulin resistance, and interfere with fatty acid metabolism inducing dyslipidemia and hyperuricemia (30). Then, the drug interaction between cyclosporine and statins, which are commonly used for hypercholesterolemia, could potentially induce rabdomiolysis (31). Consequently, cyclosporine should be used with caution in psoriatic patients with features of the metabolic syndrome. Moreover, the presence of an established CKD is a contraindication for cyclosporine use. Acitretin could also favor hypertriglyceridemia and/or hypercholesterolemia, although severe cases are rare (30). PUVA and narrowband UVB therapy are not expected to cause significant changes in metabolic parameters (32), but are impractical for many patients.

**Table 1:** **Systemic treatments for psoriasis could negatively affect the components of metabolic syndrome**.

	MTX	CsA	Acitretin	Anti-TNF-α	Ustekinumab
Hyperlipidemia	No	Yes	Yes	No	No
Hypertension	No	Yes	No	No	No
Obesity	No	No	No	Yes	No
Diabetes	No	Yes	No	No	No
NAFLD	Yes	No	No	No	No
Decreased renal function	Yes	Yes	No	No	No

*NAFLD, non-alcoholic fatty liver disease*.

Generally, biological therapies do not negatively affect metabolic parameters as conventional treatments can do. However, clinically significant dyslipidemia has been occasionally reported in patients receiving TNF-α antagonists, but this is not a common issue in clinical practice (33). A body weight gain could occur in patients treated with TNF-α antagonists (34, 35). Weight changes are induced mainly by fat mass gain in patients with psoriasis receiving TNF-α antagonists (34). By contrast, ustekinumab is not associated to body weight increase in patients with chronic plaque psoriasis (36).The effects of anti-TNF therapy on insulin sensitivity is controversial (37, 38). The effects of TNF-α inhibitors on glycemic parameters and insulin resistance in patients with psoriasis have been addressed mostly in small studies by means of the Homeostasis Model Assessment (HOMA) and the Quantitative Insulin Sensitivity Check Index (QUICKI), two widely used non-invasive surrogate markers of insulin resistance and sensitivity, respectively. A randomized, double-blind study in 12 psoriatic patients at high risk of developing type 2 diabetes mellitus failed to see a significant effect of a 2-week treatment with etanercept on insulin secretion and sensitivity (39). In contrast, a 24-week study in nine patients with stable active plaque psoriasis treated with etanercept found a significant reduction in insulin plasma levels, with a significant improvement in insulin resistance as suggested by the decrease in the HOMA index (40). No significant changes in insulin sensitivity or in the levels of fasting blood glucose were seen in a study in 18 patients with psoriasis after 12 weeks of treatment with adalimumab (41). Solomon et al. found that patients with rheumatoid arthritis or psoriasis receiving TNF-inhibitors had lower risk of developing diabetes mellitus compared with other treatments (42).

Biologics targeting the IL-12/23 pathway have proven to be highly effective in psoriasis, but their safety on CDV risk is still a matter of controversy. Indeed, briakinumab was withdrawn from clinical development because of an imbalance of major CDV events in the treatment group compared to controls (43). Consequently, there have been similar concerns over the safety of ustekinumab, although data from international post-marketing registry does not show any risk (44). To date, the Food and Drug Administration has not issued changes to the prescribing information for ustekinumab related to CDV risk. It could be also considered that drugs used mainly in the treatment of arterial hypertension including beta-blockers and ACE-inhibitors may worsen psoriasis in some patients (45). Finally, patients with moderate to severe psoriasis are candidate to interventions aimed to reduce their CDV risk including hypo-caloric diet, regular physical activity, and smoking cessation. Low calorie diet inducing a moderate weight loss (i.e., 5–10% of body weight) increases the responsiveness of obese patients to any systemic treatments (46–48). Smoking habit has been associated to onset and worsening of psoriasis, and smoking cessation can positively affect the disease course (49). Patients with psoriasis exhibit decreased levels of physical activity, possibly for both psychological and physiological reasons (50). Regular physical activity may lower the risk of incident psoriasis and have also a beneficial effect on the natural course of the disease influencing the severity as well as metabolic comorbidities (51).

From a clinical prospective, the understanding of the patients in the context of these comorbidities is very important to ensure that treatment is tailored to meet the individual patient needs.

## Conflict of Interest Statement

Paolo Gisondi has been a consultant and/or speaker for Abbott, Janssen, Leo-Pharma, Lilly, Merck Sharp & Dohme, Novartis, and Pfizer. Arturo Galvan has nothing to declare. Luca Idolazzi has nothing to declare. Giampiero Girolomoni has been a consultant, investigator, and/or speaker for AbbVie, Almirall, Amgen, Boeringher-Ingelheim, Celgene, Galderma, GlaxoSmithKline, Eli-Lilly, Janssen, Leo-Pharma, Merck Serono, Merck Sharp & Dohme, Novartis, Otsuka, Pfizer, and Shiseido.
